# Acute Obstructive Hydrocephalus Due to Posterior Reversible Encephalopathy Syndrome Successfully Treated With Continuous Ventricular Drainage: A Case Report

**DOI:** 10.7759/cureus.80803

**Published:** 2025-03-18

**Authors:** Raimu Ogino, Daisuke Yamamoto, Shintaro Furuya, Katsunobu Teshigahara, Jiro Kamiyama

**Affiliations:** 1 Emergency Medicine, Saitama Red Cross Hospital, Saitama, JPN; 2 Anesthesia and Critical Care, Saitama Medical University, Saitama Medical Center, Kawagoe, JPN; 3 Intensive Care Unit, Saitama Red Cross Hospital, Saitama, JPN

**Keywords:** blood pressure, obstructive hydrocephalus, outcome, posterior reversible encephalopathy syndrome (pres), ventricular drainage

## Abstract

Posterior reversible encephalopathy syndrome (PRES) is an acute-onset neurological disorder characterized by various symptoms, including headache, visual disturbances, and seizures. The causes of PRES include hypertension, eclampsia, sepsis, renal failure, autoimmune diseases, and the use of chemotherapeutic agents or immunosuppressants. Although the exact pathophysiology remains unclear, it is thought to involve vasogenic edema and endothelial dysfunction. PRES generally has a favorable prognosis, and with appropriate early intervention, neurological function typically recovers without residual deficits. However, in severe cases or when diagnosis and treatment are delayed, progressive cerebral edema can lead to complications such as cerebral hemorrhage or infarction, potentially resulting in irreversible neurological deficits. In this report, we present a case of severe PRES complicated by obstructive hydrocephalus, in which early diagnosis and treatment resulted in a favorable clinical outcome without neurological deficits.

A 47-year-old woman with a history of hypertension, dyslipidemia, and rheumatoid arthritis, which was in remission, had been prescribed 40 mg of nifedipine daily for hypertension. However, her medication compliance was poor, and she had discontinued antihypertensive treatment on her own approximately six months prior. Although her home-monitored systolic blood pressure (SBP) had previously averaged around 170 mmHg, it gradually increased, reaching over 200 mmHg approximately two months before admission. Two days prior to admission, she began experiencing headaches and gait disturbances, followed by seizures and impaired consciousness, prompting her emergency transport to our emergency department.

Brain computed tomography (CT) and magnetic resonance imaging (MRI) revealed severe cerebral edema, most notably in the cerebellum, resulting in obstruction of the fourth ventricle and enlargement of the third and lateral ventricles, consistent with obstructive hydrocephalus. Emergency ventricular drainage was performed to prevent brain damage from elevated intracranial pressure. She was subsequently managed in the intensive care unit (ICU) with blood pressure control, sedation, and mechanical ventilation. Follow-up brain CT on day five showed improvement of cerebral edema and resolution of hydrocephalus.

Her consciousness improved, and she regained the ability to walk independently, allowing discharge to her home.

Although PRES generally has a good prognosis, severe cases with extensive cerebral edema may have poor outcomes. In this case, the edema was particularly prominent in the cerebellum, causing cerebrospinal fluid (CSF) outflow obstruction, which likely led to obstructive hydrocephalus. However, even in severe cases, early diagnosis and timely intervention can result in favorable clinical outcomes. In PRES, persistent severe cerebral edema may lead to obstructive hydrocephalus. In this case, early ventricular drainage and blood pressure management contributed significantly to a favorable neurological outcome. When diagnosing PRES, it is important to consider the possibility of hydrocephalus secondary to cerebral edema and to prioritize early and appropriate management of blood pressure and intracranial pressure.

## Introduction

The posterior reversible encephalopathy syndrome (PRES) is an acute-onset neurological disorder characterized by a variety of symptoms, including headache, visual disturbances, and seizures [1,2]. The causes of PRES include hypertension, eclampsia, sepsis, renal failure, autoimmune diseases, and the use of chemotherapeutic agents or immunosuppressants [2]. The exact pathophysiology of PRES is not fully understood but is thought to involve mechanisms such as hypertension and endothelial dysfunction. According to the vasogenic edema hypothesis, when blood pressure exceeds the upper limit of cerebral autoregulation, cerebral blood flow increases, leading to increased vascular permeability and subsequent vasogenic edema. In contrast, the endothelial dysfunction hypothesis suggests that factors such as eclampsia, sepsis, or drug-induced vascular injury cause endothelial damage, resulting in increased vascular permeability and edema formation [3]. With appropriate and early treatment, neurological function typically recovers without residual deficits, and the prognosis is generally favorable. However, delays in treatment or the progression of cerebral edema may lead to poor outcomes [4]. PRES predominantly affects the occipital lobe, but findings can also be observed in the brainstem, cerebellum, and spinal cord. If edema occurs in the cerebellum or brainstem, which are part of the cerebrospinal fluid (CSF) outflow pathways, it may lead to obstructive hydrocephalus due to the narrowing of these pathways. If diagnosis and treatment are delayed, there is a risk of brain damage due to increased intracranial pressure (ICP). If diagnosis and treatment are delayed, the risk of brain damage due to increased ICP rises.

In this report, we present a severe case of PRES with significant cerebral edema and obstructive hydrocephalus, successfully treated with ventricular drainage, resulting in a favorable outcome.

## Case presentation

A 47-year-old woman with a history of hypertension, dyslipidemia, and rheumatoid arthritis, which was in remission, had been taking 40 mg of nifedipine for hypertension. She had been monitoring her blood pressure at home, and her systolic blood pressure (SBP) had remained approximately 170 mmHg. However, she had discontinued the medication on her own about six months prior, leading to an increase in SBP to over 200 mmHg for about one month. She initially developed headaches and gait disturbances two days before her visit, followed by seizures and impaired consciousness, prompting her emergency transport to the emergency department of our critical care center. Upon arrival at the emergency department, her Glasgow Coma Scale (GCS) score was 6, with eye 1, verbal 1, and motor 4 (E1V1M4). She exhibited left conjugate gaze deviation and generalized tonic seizures, predominantly affecting the left upper limb, but her pupillary findings were normal. Her blood pressure was significantly elevated at 201/147 mmHg, with a heart rate of 94 beats per minute (bpm). She was initially treated with 10 mg of diazepam intravenously, which temporarily suppressed the seizures, but they recurred within minutes. Intravenous levetiracetam (1,000 mg) was given but did not achieve sufficient seizure control. The condition was deemed to have progressed to refractory status epilepticus (RSE). As she progressed into RSE, deep sedation management and mechanical ventilation were initiated, and third-line therapy with continuous intravenous anesthesia was started. A continuous infusion of propofol and fentanyl was administered, aiming for seizure suppression for 48 hours.

**Figure 1:**
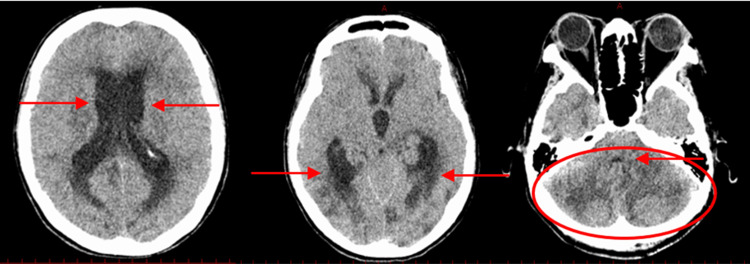
Non-contrast Brain Computed Tomography (CT) on Initial Examination Obstructive hydrocephalus with dilation of the third and lateral ventricles (red arrow). Narrowing of the fourth ventricle (red arrow) and hypodense areas in the cerebellum, along with posterior brain edema (red circle).

A brain computed tomography (CT) scan showed multiple low-density areas in the cerebrum and cerebellum, cerebral edema, and enlargement of the third ventricle due to narrowing of the fourth ventricle, indicating obstructive hydrocephalus (Figure 1).

**Figure 2:**
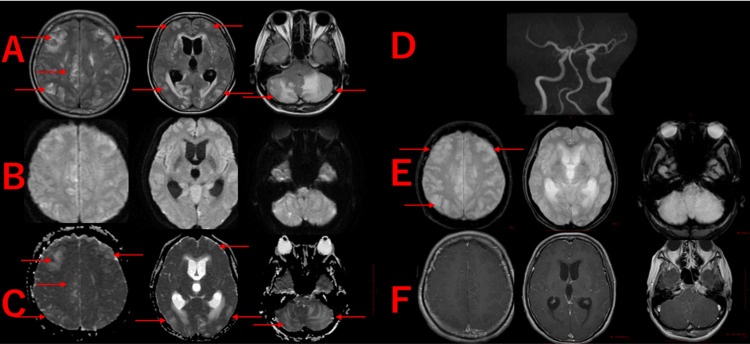
Brain Magnetic Resonance Imaging (MRI) on Initial Examination A: Fluid-attenuated inversion recovery (FLAIR) - High-signal areas are observed mainly in the cerebellum and brainstem, extending to the cerebral cortex and white matter (red arrow), B: Diffusion-weighted imaging (DWI) - No high-signal areas are detected, C: Apparent diffusion coefficient (ADC) - High-signal areas are observed in the same regions as on FLAIR, indicating vasogenic edema (red arrow), D: Magnetic resonance angiography (MRA) - No abnormalities are observed in the cerebral arteries, E: T2-weighted imaging - High-signal areas are noted (red arrow), F: Gadolinium-enhanced T1-weighted imaging (Gd-enhanced T1WI) - No contrast-enhancing tumors or thrombosis are observed.

Brain magnetic resonance imaging (MRI) revealed multiple high-signal areas on fluid-attenuated inversion recovery (FLAIR) imaging, symmetrically affecting the cerebellum and brainstem, extending from the cerebral cortex to the white matter (Figure 2A). Diffusion-weighted imaging (DWI) showed no high-signal areas, and there were no findings suggestive of cerebral infarction (Figure 2B). The apparent diffusion coefficient (ADC) showed high-signal areas, indicating findings consistent with vasogenic edema (Figure 2C). Magnetic resonance angiography (MRA) showed no abnormalities in the cerebral arteries (Figure 2D). T2-weighted imaging also showed high signal intensity in the same region as FLAIR (Figure 2E). Contrast-enhanced MRI was performed to rule out a brain tumor and thrombosis, and neither were found (Figure 2F).

Laboratory tests obtained at the time of admission revealed an elevated D-dimer level. However, there were no findings suggestive of thrombosis or bleeding. The tests revealed anemia, an elevated creatinine level, and an increased D-dimer, indicating acute kidney injury and thrombocytopenia (Table 1).

**Table 1:** Blood test results on initial examination.

Variables	Test values	Reference ranges	Units
Sodium	146	138-145	mEq/l
Potassium	3.8	3.6-4.8	mEq/L
Glucose	198	70-110	mg/dl
Total bilirubin	0.4	0.4-1.5	mg/dl
Direct bilirubin	0.1	0.1-0.4	mg/dl
Urea	42.6	10-55	mg/dl
Creatinine	2.02	0.7-1.4	μmol/l
Aspartate transaminase	43	5-40	U/l
Alanine transaminase	16	5-40	U/l
White blood cell count	9.37	4.0-10.0	10^3^/µl
Red blood cell count	4.23	4.5-4.8	10^6^/µl
Hemoglobin	7.6	11.6-15.5 (Women)	g/dl
Platelet count	69	140-450	10^3^/µl
Prothrombin time (international normalized ratio)	0.76	0.8-1.2	-
Activated partial thromboplastin time	26.1	26.0-36.0	sec
D-dimer	3.4	<0.6	µg/ml

The elevation was suspected to be due to microthrombus formation caused by endothelial damage from hypertension.

Due to severe cerebral edema and the risk of brain herniation, lumbar puncture was not performed; instead, emergency ventricular drainage was performed for ICP control and CSF sampling. CSF analysis revealed only a mild elevation in cell count (Table 2).

**Table 2:** Cerebrospinal fluid (CSF) test results on initial examination. HSV-DNA PCR: Herpes simplex virus deoxyribonucleic acid polymerase chain reaction

Variables	Test values	Reference ranges	Units
Appearance	Cloudy and red	Clear, colorless	-
Total Cell Count	42	0-5	cells/µl
Protein	37	15-45	mg/dl
Glucose	68	40-70	mg/dl
HSV-DNA PCR	Negative	Negative	-

There were no findings of bacterial meningitis or encephalitis, such as an elevated cell count or decreased glucose levels. The mild elevation in cell count was considered to be due to the effects of a traumatic tap. The external ventricular drain was set at +15 cmH₂O based on the external reference point (foramen of Monro), allowing for continuous CSF drainage. The causes of hydrocephalus were evaluated, including neoplastic lesions, infectious diseases such as meningitis and encephalitis, and hemorrhage. However, based on the results of the aforementioned tests, none of these conditions were considered likely. The obstructive hydrocephalus in this case was considered to be caused by CSF outflow obstruction due to cerebellum-centered cerebral edema. Based on the clinical course and examination findings, she was diagnosed with PRES.

**Figure 3:**
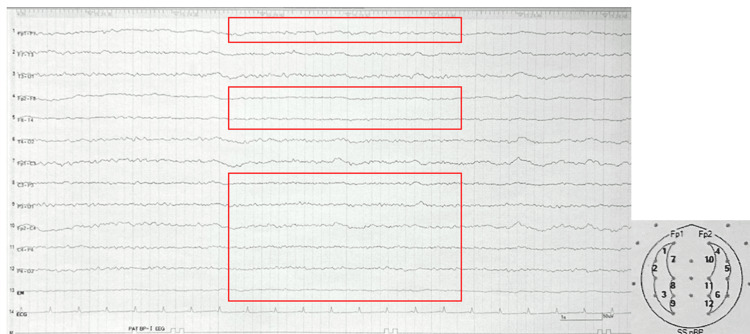
Electroencephalography on the Second Day of Hospitalization Scalp electrodes were placed according to the International 10-20 System during the Electroencephalography (EEG) examination. Recorded under continuous infusion of propofol and fentanyl. Widespread low-amplitude slow waves were observed (red square), with no epileptiform discharges detected.

She was transferred from the emergency department to the intensive care unit (ICU), where 48 hours of deep sedation were initiated for blood pressure control and management of status epilepticus. Since rapid blood pressure reduction carries a risk of cerebral ischemia [5], the target reduction was adjusted to prevent a decrease of more than 25% from the initial blood pressure. Nicardipine hydrochloride was used to rapidly lower SBP to a target of 160 mmHg, followed by a gradual reduction to 140 mmHg over 24 hours. Continuous intravenous administration of fentanyl and propofol, along with mechanical ventilation, was performed to maintain deep sedation. Considering the possibility of epileptic seizures, levetiracetam (1,000 mg/day) was continued. Electroencephalography (EEG) was performed to confirm the achievement of burst suppression, allowing evaluation of sustained seizure control (Figure 3).

**Figure 4:**
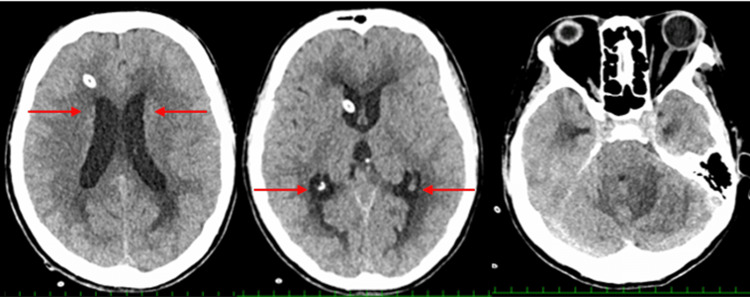
Non-Contrast Brain Computed Tomography (CT) on the Fifth Day of Hospitalization Follow-up brain CT showed a reduction in low-density areas, along with improvements in brain edema and hydrocephalus (red arrow).

Deep sedation was then continued for 48 hours [6]. An EEG was recorded to confirm the achievement of burst suppression and to evaluate whether seizure control was maintained (Figure 4). On the fifth day of hospitalization, follow-up brain CT showed a reduction in the size of the third ventricle, indicating improvement of hydrocephalus (Figure 4).

**Figure 5:**
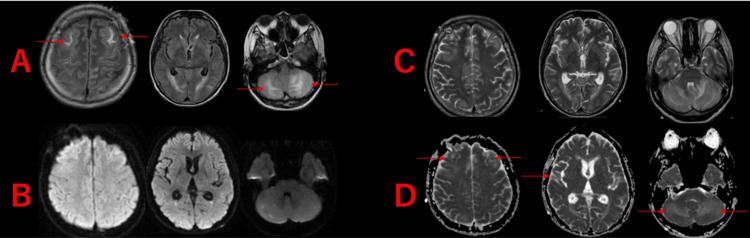
Brain Magnetic Resonance Imaging (MRI) on the Sixth Day of Hospitalization A: Fluid-attenuated inversion recovery (FLAIR), B: Diffusion-weighted imaging (DWI), C: T2-weighted imaging (T2WI), D: Apparent diffusion coefficient (ADC) High-signal areas observed on FLAIR and ADC at the time of admission have improved (red arrow).

After discontinuation of sedatives, her level of consciousness was GCS 4 (E3VtM1). Brain MRI performed on hospital day 6 also showed a reduction in cerebral edema (Figure 5).

On the seventh day of hospitalization, the ventricular drain was removed, and mechanical ventilation was discontinued on the eighth day. She was transferred out of the ICU on the tenth day of hospitalization. Antihypertensive treatment with 4 mg of candesartan and 2.5 mg of amlodipine besylate was continued. The patient was transferred to a rehabilitation hospital on the 57th day of hospitalization and was later discharged home without any residual deficits, returning to her pre-admission condition.

## Discussion

PRES was first described by Hinchey et al. in 1996 [1]. The disease is characterized by acute-onset reversible cerebral edema associated with conditions such as hypertension, immunosuppressant use, and renal dysfunction. The main clinical symptoms include headache, seizures, impaired consciousness, and visual disturbances.

The characteristic imaging findings include subcortical white matter lesions on FLAIR and T2-weighted MRI [4, 5, 7, 8], but there are no standardized diagnostic criteria for PRES. Thus, diagnosis is based on a comprehensive evaluation of clinical symptoms, patient background factors, and neuroimaging findings, with differentiation from conditions such as cerebral infarction, reversible cerebral vasoconstriction syndrome, infections, and tumors [2, 8].

The pathophysiology of PRES is thought to involve endothelial dysfunction and impaired cerebral autoregulation [2, 4]. Endothelial injury caused by immunosuppressive therapy, renal failure, or sepsis leads to increased vascular permeability, resulting in cerebral edema. Additionally, a sudden increase in blood pressure exceeding the autoregulatory capacity of the brain leads to vasodilation, increased permeability of the blood-brain barrier, and vasogenic edema [4].

PRES predominantly affects the posterior circulation. The vertebrobasilar system has less sympathetic innervation and lower autoregulatory capacity than the internal carotid system, making it more susceptible to hypertensive damage [9]. However, when PRES extends to the cerebellum, brainstem, or basal ganglia, it can cause CSF outflow obstruction due to cerebral edema [3, 10]. In such cases, CSF outflow obstruction due to cerebral edema may lead to increased ICP, impaired consciousness, and a potentially fatal clinical course [10]. Therefore, evaluating the extent of lesions in the posterior circulation and initiating appropriate early treatment interventions are crucial when diagnosing PRES.

In this case, cerebellum-centered cerebral edema likely caused narrowing of the CSF outflow pathways, resulting in obstructive hydrocephalus. Therefore, emergency ventricular drainage was performed promptly to manage intracranial hypertension. The current case was characteristic because it involved PRES complicated by obstructive hydrocephalus. PRES usually resolves spontaneously with appropriate blood pressure management; however, cases with CSF outflow obstruction, as seen in our patient, are rare. Reports of PRES cases requiring ventricular drainage are limited [9-12]. In this case, early ventricular drainage and antihypertensive treatment contributed to a favorable outcome.

PRES generally has a favorable prognosis, often resolving without residual neurological deficits. Factors associated with poor outcomes include persistent hypertension leading to progressive cerebrovascular injury and cerebral edema extending to the brainstem or cerebellum, causing impaired consciousness and respiratory depression. Additionally, prolonged hydrocephalus can lead to progressive ventricular enlargement and permanent neurological deficits [10]. If early MRI diagnosis is not performed and treatment is delayed, appropriate blood pressure control and ventricular drainage may not be initiated in time, which increases the risk of irreversible neurological damage. Discontinuation of any causative medications is also an important aspect of management. This case highlights the risk of obstructive hydrocephalus in PRES and underscores the importance of appropriate management. In particular, careful monitoring for hydrocephalus progression and timely therapeutic interventions, including ventricular drainage, can lead to improved neurological outcomes in PRES.

## Conclusions

In PRES, progressive intracranial hypertension can lead to CSF outflow obstruction, potentially resulting in irreversible neurological deficits. However, the factors or patient backgrounds associated with severe cases or susceptibility to obstructive hydrocephalus remain unclear, and further accumulation of clinical data is required.

When diagnosing PRES, clinicians should pay close attention not only to the extent of lesions within the posterior circulation but also to changes in CSF dynamics. Prompt ICP management, including ventricular drainage, should be considered when necessary to prevent neurological deterioration.
